# A finite element analysis on comparing the stability of different posterior fixation methods for thoracic total en bloc spondylectomy

**DOI:** 10.1186/s13018-020-01833-0

**Published:** 2020-08-12

**Authors:** Yun Liang, Yuanwu Cao, Zhiguo Gong, Chang Jiang, Lixia Jin, Zheng Li, Zixian Chen, Chun Jiang, Xiaoxing Jiang

**Affiliations:** 1 Department of Orthopaedics, Shanghai Zhongshan Hospital, Fudan University, 180 Fenglin Road, Shanghai, 200032 People’s Republic of China; 2 Department of Orthopaedics, Wan bailin Branch, The First Hospital of Shanxi Medical University, 2 Zhongfang Road, Wanbailin District, Taiyuan, 030024 Shanxi Province People’s Republic of China

**Keywords:** Thoracic vertebra, Thoracic cage, TES, Spinal stability, Finite element analysis

## Abstract

**Objective:**

To compare the spinal stability with different fixation methods after thoracic TES using finite element analysis

**Methods:**

The spinal finite element model was established from a healthy volunteer, and the validity was verified. The models of T8 thoracic total en bloc spondylectomy (TES) with and without artificial vertebral body were established combination with different fixation methods: the first was long segment fixation with fixed segments T5–7, T9–11; the second was short segment fixation with fixed segments T6–7, T9–10; the third was modified short segment with a pair of vertebral body screws on T7 and T9 added on the basis of short segment fixation. The motions of each model in standing state were simulated in software. The range of motion (ROM) and internal fixation stress changes were analyzed.

**Results:**

When anterior support was effective, the three fixation methods could effectively maintain the stability of the spine. However, when anterior support failed, the ROM of the long segment fixation group and the short segment fixation group in the flexion-extension directions was significantly higher than that of when the anterior support existed, while the modified short segment fixation group had no significant changes. Meanwhile, the stress of internal fixation in the long segment fixation group and the short segment fixation group were greatly increased. However, there were no significant changes in modified short segment fixation group.

**Conclusion:**

After TES, the presence of the thoracic cage gives partial anterior stabilization. When the anterior support failed, the modified short segment fixation method can provide better stability.

## Background

With the deepening understanding of spinal tumors, especially the establishment of the Weinstein-Boriani-Biagini (WBB) surgical staging and the Tomita classification system [1, 2], the continuous development of spinal fixation and reconstruction instruments, the techniques used for spinal tumor surgery have made great progress. The surgical treatment of spinal tumors has been no longer limited to simple laminectomy and piecemeal resection, and the TES have been well performed, which significantly improved the treatment of spinal tumors. However, reconstruction of spinal stability after TES is always the focus of attention and one of the prerequisites for patients to obtain a good prognosis.

Compared with partial vertebral resection, reconstruction of spinal stability is more difficult after TES due to removal of the affected vertebral body, posterior column structure, and the associated stabilizing soft-tissue structures [3]. Because of the limitations of the surgical approach, the most common reconstruction method currently is the anterior support with titanium mesh or artificial vertebral body combined with posterior pedicle screw fixation [4]. However, the number of fixed segments of the spine after resection has been the focus of debate. Currently, the commonly posterior fixation method is bilateral pedicle screw fixation at least two segments above and below the resection segment [5–7]. There are also some researchers who chose to increase the lateral fixation of the vertebral body on this basis [8]. According to the results of biomechanical experiments, Oda et al. recommended anterior fixation combined with posterior short segment fixation [9], whereas Disch et al. believe that the multi-segment posterior instrumentation can ensure the fixation strength while the short segment fixation cannot provide sufficient stability even if the anterior lateral plate is included [3]. Therefore, there are still some disputes on the reconstruction method. Current biomechanical experiments are all performed in vitro, in addition, the presence of the anterior thoracic cage can increase the stability of the thoracic spine [10]. Therefore, we consider whether the number of fixed segments can be reduced after thoracic TES.

The aim of this study was to compare the stability of the spine and the stress distribution of the instrumentation in three different reconstruction methods after thoracic TES using finite element analysis, and to provide guidance for clinical application.

## Materials and methods

### Establishment and verification of a thoracic spine finite element model after T8 TES

The study was approved by the Ethics Committee of Zhongshan Hospital, Fudan University, and our approval number is B2019-220R. The informed consent was given to the volunteer at the beginning of the study. The volunteer was a 45-year-old healthy male with a height of 177 cm and a normal body mass index (BMI). And X-ray was used to exclude spinal diseases including deformity and fracture of the spine. In this study, T8 was selected as resected segment. The computed tomography (CT) scan of the whole thoracic spine and thoracic cage was performed on the volunteer. Then, the images of T5–T11 including thoracic cage were obtained, and the slice thickness was 0.5 mm. The image data were exported as Digital Imaging and Communications in Medicine (DICOM) format. The DICOM images were imported into the Mimics 20.0 software (Materialise, Leuven, Belgium) to obtain the vertebral body and rib model in STL format, which was transformed into a solid model by the Geomagic 12.0 software (Geomagic, North Carolina) and combined with the intervertebral disc model. At the end, a 3-dimensional (3D) finite element model was established including a normal T5 to T11 thoracic spine without T8 and thoracic cage. The material parameters were selected according to the previously published literatures [11–13] (Table 1).

**Table 1:** Material parameters of different materials in the finite element model

	Young’s modulus (MPa)	Poisson’s ratio
Cortical bone	12,000	0.3
Cancellous bone	100	0.2
Posterior bony unit	3500	0.25
Cartilage endplate	4000	0.3
Annulus fibrosus	4.2	0.45
Nucleus pulposus	1.0	0.4999
Titanium alloy	108,000	0.3

To verify the feasibility of the finite element model, the range of motion of T9–T11 was measured. The inferior endplate of T11 was assumed to be completely fixed, so that the T11 vertebral body did not have any movement. 7.5 Nm of torque was applied to the T9 in six directions including flexion, extension, bending to the left, bending to the right, rotation to the left, and rotation to the right. The ROM values of T9–10 and T10–11 were recorded and compared with previously published results (Table 2).

**Table 2:** Comparison of the ROM of T9–10 and T10–11 with published study (unit: degree)

	T9–10		T10–11	
	Present study	Published study	Present study	Published study
Flexion	3.52	2.51–4.50	2.876	2.03–4.14
Extension	3.66	2.72–4.40	2.727	2.03–4.30
Bending to the left	2.91	2.85–6.06	2.108	2.99–5.24
Bending to the right	2.95	2.87–5.89	2.13	2.93–5.13
Rotation to the left	8.17	3.61–6.55	6.09	3.10–4.79
Rotation to the right	7.32	3.65–6.64	5.96	3.07–4.77

### Establishment of fixation model containing artificial vertebral body after T8 TES

**Fig. 1:**
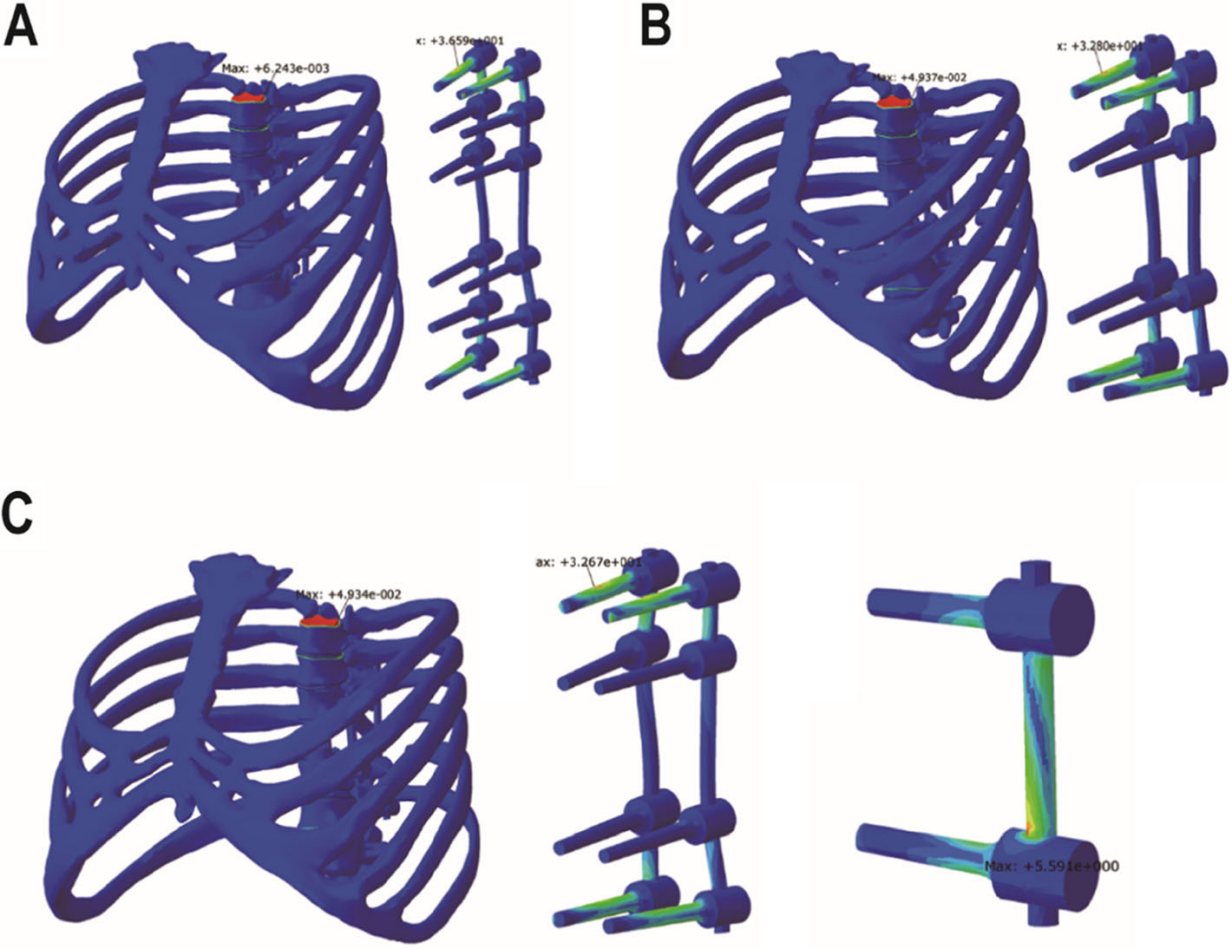
Three fixation models containing artificial vertebral body. **a** Long segment fixation. **b** Short segment fixation. **c** Modified short segment fixation

After the finite element model was verified, pedicle screw, rod, and artificial vertebral body model were created and combined with T5 to T11 model by the UG 10.0 software (Siemens PLM Software, GER). The length and diameter of the screw were 40 mm and 5.5 mm, respectively. The artificial vertebral body had an inner diameter of 16 mm and an outer diameter of 20 mm. The diameter of the rod was 5.5 mm. According to the different fixation methods, the models were divided into three groups (Fig. 1). Group A referred to bilateral posterior fixation with 12 pedicle screws (in T5–T7, T9–T11) and was defined as long segment fixation. Group B referred to bilateral posterior fixation with 8 pedicle screws (in T6, T7, T9, and T10) and was defined as short segment fixation. Group C referred to a combination of bilateral posterior fixation with 8 pedicle screws (in T6, T7, T9, and T10) and 2 lateral vertebral body screws (in T7 and T9) which were connected by a rod, and was defined as modified short segment fixation.

### Establishment of fixation model without artificial vertebral body after T8 TES

**Fig. 2:**
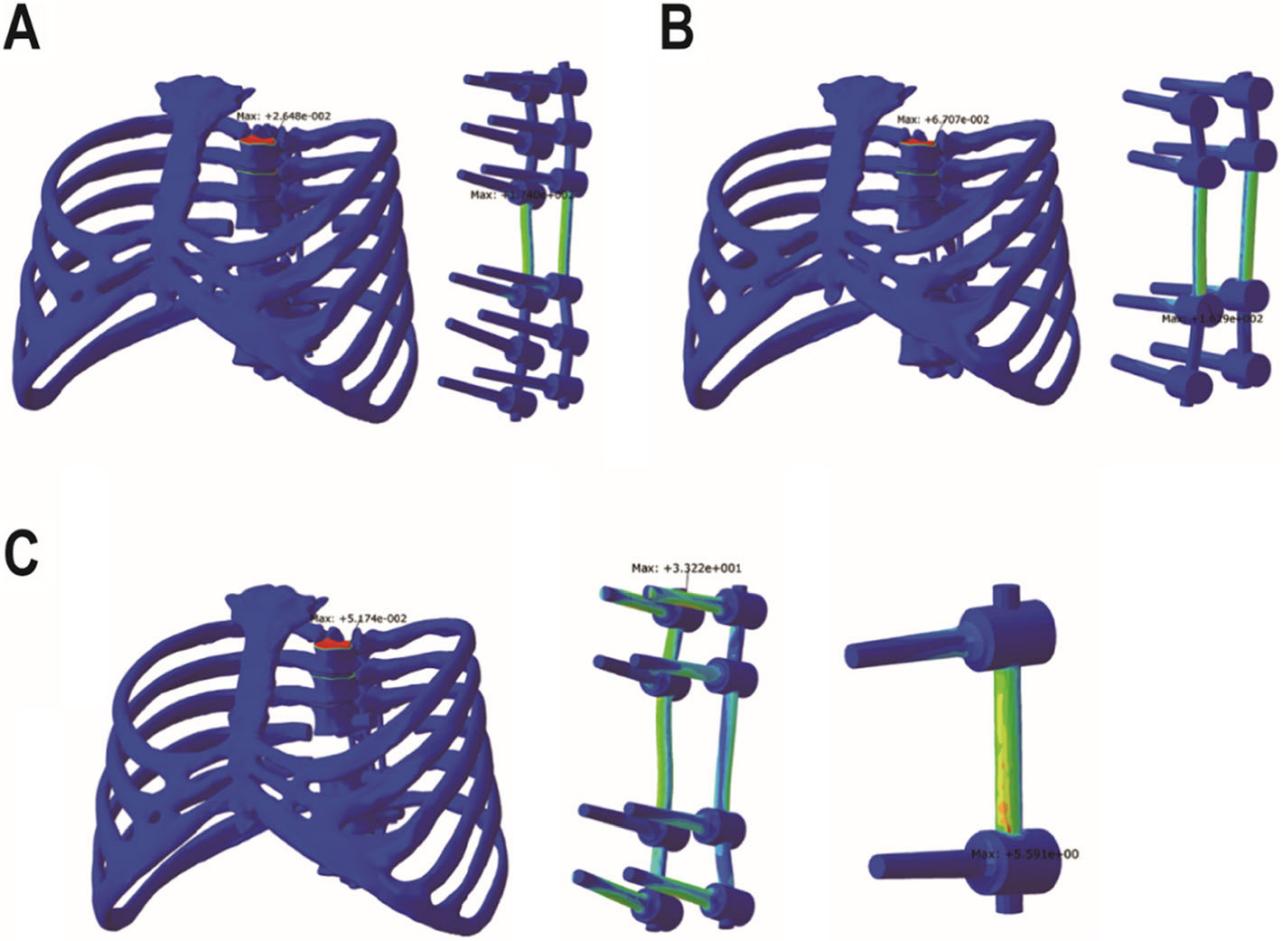
Three fixation models without artificial vertebral body. **a** Long segment fixation. **b** Short segment fixation. **c** Modified short segment fixation

In order to simulate the complication of anterior support failure after TES, the finite element models of three different fixation methods without artificial vertebral body were established (Fig. 2). The fixation methods were the same as above and were recorded as three groups, a, b, and c, respectively.

### Assessment of indicators

The data of 6 models were imported into the Hypermesh 14.0 software (Altair, USA) and Abaqus 6.14 software (Dassault Systèmes, USA), and the material parameters were added. The inferior endplate of T11 was assumed to be completely fixed, and there is no displacement in the interface between the screw and the bone. A 500 N axial load was pre-applied to the models to simulate the stress of the spine in standing position, and 7.5 Nm of torque was applied to the models in six direction including flexion, extension, bending to the left, bending to the right, rotation to the left, and rotation to the right. The ROM was calculated as the angular displacement caused by applied force in all directions, which was directly analyzed by the software. By detecting the stress on each position of the internal fixation under the applied force, a stress distribution map was drawn by the software to obtain the most concentrated part of the stress. The maximum stress was compared with the yield strength of the internal fixation material. There is no statistical analysis of the data in this study, since there was only one research object.

## Result

### Fixation models containing artificial vertebral body

**Fig. 3:**
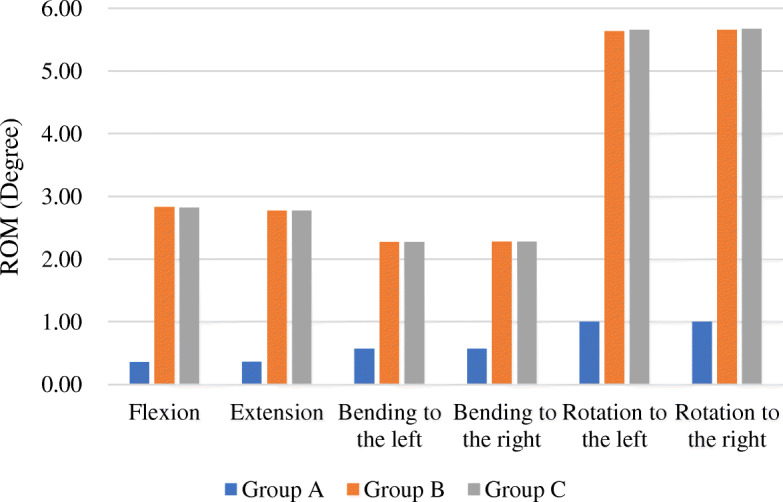
The ROM of three models containing artificial vertebral body

When the model contained artificial vertebral, that was, the anterior support was available, three fixation methods all can effectively maintain the stability of the spine. Compared to the other two groups, the long segment fixation was more rigid. The ROMs of group A in each direction had dramatically decreased by 75.0 to 87.4% than the other two groups, especially in flexion and extension. In addition, the ROM of group B and group C was not much different. The histogram shows the differences between the three fixation methods more intuitively (Fig. 3).

**Fig. 4:**
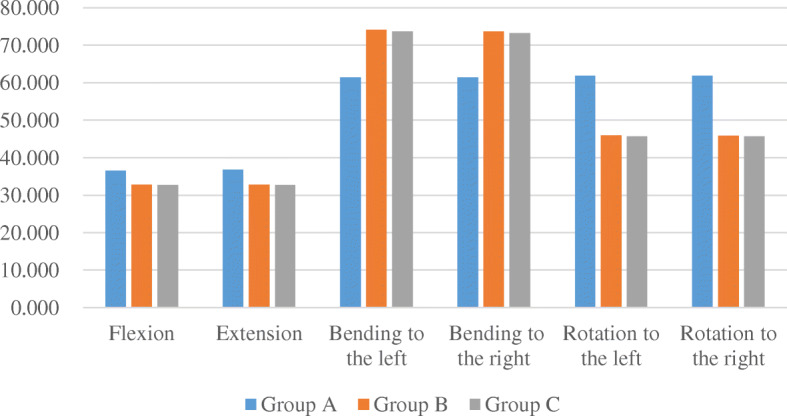
The maximum stress of three models containing artificial vertebral body

In terms of the stress of screws and rods, the maximum stress of group A during flexion and extension was slightly stronger than the other two groups but seemed smaller than the other two groups when bending to the left or right, and was significantly stronger (26%) than the other two groups when rotating (Fig. 4). The maximum stress of the three groups in all directions was almost located at both ends of the fixed segment. And the maximum stress of three groups in all directions was less than the yield strength of internal fixation material.

### Fixation models without artificial vertebral body

When the model did not contain the artificial vertebral body, that was, when the anterior support failed, the ROM of the long segment fixation and the short segment fixation in the flexion and extension directions was obviously higher than that of the models containing the artificial vertebral body (Table 3). However, there were no obvious changes in the modified short segment fixation group in any direction.

**Table 3:** Comparison of the ROM of three groups with and without artificial vertebral body (unit: degree)

	Group A	Group a	Δ	Group B	Group b	Δ	Group C	Group c	Δ
Flexion	0.358	1.517	324.16%	2.829	3.896	37.72%	2.827	2.964	4.86%
Extension	0.360	1.521	322.37%	2.771	3.843	38.66%	2.770	2.908	4.99%
Bending to the left	0.568	0.571	0.52%	2.271	2.288	0.76%	2.272	2.286	0.58%
Bending to the right	0.568	0.571	0.50%	2.278	2.294	0.73%	2.279	2.293	0.60%
Rotation to the left	1.002	1.403	39.97%	5.638	5.976	6.00%	5.655	5.747	1.63%
Rotation to the right	1.002	1.403	39.97%	5.657	5.993	5.95%	5.673	5.764	1.60%

Compared with the models containing the artificial vertebral body, the maximum stress of pedicle screws and rods in groups a and b were significantly increased by about 375% and 395% in flexion and extension directions, and 57% and 89% in rotation, respectively (Table 4). However, the stress did not change much when bending left and right. As for the modified short segment fixation group, the maximum stress of the pedicle screws and rods in all directions had no significant change compared with the models containing the artificial vertebral body. In addition, the stress of vertebral body screws was increased by 900%, 80%, and 210% in flexion and extension, bending, and rotation, respectively, but the values of stress were much less than that of groups a and b. In groups a and b, the maximum stress location in all directions changed from the two ends of the internal fixation to the near resected segment. However, in group c, there was no significant change in the distribution of the maximum stress. In addition, the maximum stress of three groups in all directions was also less than the yield strength of internal fixation material.

**Table 4:** Comparison of the maximum stress of three groups with and without artificial vertebral body

	Group A	Group a	Δ	Group B	Group b	Δ	Group C	Group c	Δ
Flexion	36.590	174.000	375.54%	32.800	162.100	394.21%	32.670	33.220	1.68%
Extension	36.750	174.500	374.83%	32.800	162.900	396.65%	32.670	33.490	2.51%
Bending to the left	61.460	62.230	1.25%	74.120	74.470	0.47%	73.720	75.020	1.76%
Bending to the right	61.380	62.170	1.29%	73.660	74.130	0.64%	73.240	74.560	1.80%
Rotation to the left	61.780	97.310	57.51%	45.910	86.760	88.98%	45.700	43.540	− 4.73%
Rotation to the right	61.780	97.370	57.61%	45.890	86.840	89.24%	45.680	43.570	− 4.62%

## Discussion

The development of the TES technique had changed the shortcomings of traditional intracapsular curettage and piecemeal resection, such as high recurrence rate and poor tumor control, which had been paid more and more attention by spinal surgeons. When TES was successfully performed, another major challenge for the surgeon was the reconstruction of spinal stability. As the support of the upper body, the stability of the spine played an extremely important role in daily life. The researches had shown that the incidence of instrumentation failure after TES was nearly 40%, including rod or screw broken and artificial vertebral body or titanium mesh subsidence etc. [6, 14]. The instrumentation failure after TES was mostly due to the instability or subsidence of anterior artificial vertebral body or titanium mesh, which can result in stress concentration on the posterior screw-rod system. For multi-segment TES, most artificial vertebral body or titanium mesh subsidence occurred even after 1 month after surgery [15]. Therefore, how to maintain the stability of the anterior column was essential after TES. In the present study, we first included the factor of thoracic cage in the reconstruction of the spinal stability after thoracic TES, and the purpose was to compare the stability of three fixation methods and determine the most suitable fixation method.

When the anterior support was effective, three fixation methods could effectively maintain the stability of the spine. But the ROM of long segment fixation in all directions was reduced by 75.0–87.4% compared with the other two groups. It means that long segment fixation achieves a rigid fixation at the expense of normal mobility of the thoracic spine. Excessively rigid fixation may result in some side effects, such as stress shielding and reduction of the quality of the bone around the screw [16]. Biologic bony fusion is very crucial for long-term stability after TES. Akamaru et al. considered that high fusion stiffness was not necessary because the purpose of using instrumentation was to achieve biologic bony fusion [17]. However, another study indicated that a reconstruction method without anterior fixation should be better for fusion [18]. The authors suggested that the stress shielding of graft bone in titanium cage with posterior screw-rod system plus anterior instrumentation was higher than that of using only posterior multi-segment pedicle screw fixation, and they believed that eight pedicle screws (2 pairs of screw above and below the resected vertebral body, respectively) were stable enough. Therefore, the effect of additional anterior fixation on the stability of the spine is still one of the controversial focus.

Anterior titanium mesh or artificial vertebral body subsidence was one of the common long-term complications after TES, especially in elderly patients with osteoporosis. Matsumoto et al. reported the occurrence of 6% titanium mesh subsidence after TES [14]. Moreover, the incidence of titanium mesh subsidence after multi-segment TES was almost 50% [15]. Therefore, we analyzed the extreme condition of complete loss of anterior support. When the anterior support failed, the long segment fixation group showed a significant increase in the ROM and maximum stress when flexion, extension, and left-right rotation compared with the presence of artificial vertebral bodies. The results in the short segment fixation group were similar. However, the modified short segment fixed group had little change in all directions. The above results indicated that when anterior support completely failed, the modified short segment fixation was more stable than the other two groups, especially in flexion and extension, the advantage was obvious. In addition, the maximum stress location of long and short segment fixation in all directions changed from the two ends of the internal fixation to the near resected segment. We supposed that the adjacent segments of the resected segment were weak points of the internal fixation system. If reinforcement was performed at this location, it would play an important role in maintaining the stability of the whole internal fixation system. Our results also confirmed this hypothesis. In the modified short segment fixation group, there was no significant change in the distribution of maximum stress. The presence of the vertebral body screw well shared the stress of the posterior-rod system.

In our study, the finite element analysis was used. With the finite element model, the structure of the spine, the material properties, and the load distribution of instrumentation under various conditions can be quantified. It can be used to analyze many problems that cannot be studied in vitro, such as the stress changes inside the research subject. However, there were still some limitations in our study. First, like most finite element studies, our research had only one research object, so that there was a lack of statistical analysis. However, according to the research by Li et al., a difference more than 20% of differences was considered “important” or “relevant” [19]. Second, some questions were simplified. For example, the displacement and friction coefficient on the surface between screw and the bone were ignored. Third, soft tissues such as muscles and ligaments were removed. In the future, we plan to design a more reasonable and rigorous prospective clinical trial and biomechanical trial to verify our results.

## Conclusion

In summary, after thoracic TES, the presence of the thoracic cage gives partial anterior stabilization. Therefore, when the anterior support was effective, all the three fixation methods can effectively maintain the stability of the spine, even the short segment fixation. But when the anterior support failed, the thoracic cage could not give adequate support. At this time, the modified short segment fixation method can provide better stability than the other two fixation methods. The vertebral body screw can effectively share the stress of the posterior screw-rod system, improve the stability of the whole instrumentation system, and reduce the number of segments that needs to be fixed.

## Data Availability

The datasets used and/or analyzed during the current study are available from the corresponding author on reasonable request.

## Abbreviations

TES: Total en bloc spondylectomy
ROM: Range of motion
WBB: Weinstein-Boriani-Biagini
BMI: Body mass index
CT: Computed tomography
DICOM: Digital Imaging and Communications in Medicine
3D: 3-dimensional
